# Defining vulnerability subgroups among pregnant women using pre-pregnancy information: a latent class analysis

**DOI:** 10.1093/eurpub/ckac170

**Published:** 2022-12-14

**Authors:** J M Molenaar, L van der Meer, L C M Bertens, E F de Vries, A J M Waelput, M Knight, E A P Steegers, J C Kiefte-de Jong, J N Struijs

**Affiliations:** Department of Quality of Care and Health Economics, National Institute for Public Health and the Environment (RIVM), Centre for Nutrition, Prevention and Health Services, Bilthoven, the Netherlands; Department of Public Health and Primary Care/Health Campus The Hague, Leiden University Medical Centre, the Hague, the Netherlands; Department of Obstetrics and Gynaecology, Erasmus MC, University Medical Centre, Rotterdam, the Netherlands; Department of Obstetrics and Gynaecology, Erasmus MC, University Medical Centre, Rotterdam, the Netherlands; Department of Quality of Care and Health Economics, National Institute for Public Health and the Environment (RIVM), Centre for Nutrition, Prevention and Health Services, Bilthoven, the Netherlands; Department of Public Health and Primary Care/Health Campus The Hague, Leiden University Medical Centre, the Hague, the Netherlands; Department of Obstetrics and Gynaecology, Erasmus MC, University Medical Centre, Rotterdam, the Netherlands; Department of Public Health and Primary Care/Health Campus The Hague, Leiden University Medical Centre, the Hague, the Netherlands; National Perinatal Epidemiology Unit, Nuffield Department of Population Health, University of Oxford, Oxford, UK; Department of Obstetrics and Gynaecology, Erasmus MC, University Medical Centre, Rotterdam, the Netherlands; Department of Public Health and Primary Care/Health Campus The Hague, Leiden University Medical Centre, the Hague, the Netherlands; Department of Quality of Care and Health Economics, National Institute for Public Health and the Environment (RIVM), Centre for Nutrition, Prevention and Health Services, Bilthoven, the Netherlands; Department of Public Health and Primary Care/Health Campus The Hague, Leiden University Medical Centre, the Hague, the Netherlands

## Abstract

**Background:**

Early detection of vulnerability during or before pregnancy can contribute to optimizing the first 1000 days, a crucial period for children’s development and health. We aimed to identify classes of vulnerability among pregnant women in the Netherlands using pre-pregnancy data on a wide range of social risk and protective factors, and validate these classes against the risk of adverse outcomes.

**Methods:**

We conducted a latent class analysis based on 42 variables derived from nationwide observational data sources and self-reported data. Variables included individual, socioeconomic, lifestyle, psychosocial and household characteristics, self-reported health, healthcare utilization, life-events and living conditions. We compared classes in relation to adverse outcomes using logistic regression analyses.

**Results:**

In the study population of 4172 women, we identified five latent classes. The largest ‘healthy and socioeconomically stable’-class [*n* = 2040 (48.9%)] mostly shared protective factors, such as paid work and positively perceived health. The classes ‘high care utilization’ [*n* = 485 (11.6%)], ‘socioeconomic vulnerability’ [*n* = 395 (9.5%)] and ‘psychosocial vulnerability’ [*n* = 1005 (24.0%)] were characterized by risk factors limited to one specific domain and protective factors in others. Women classified into the ‘multidimensional vulnerability’-class [*n* = 250 (6.0%)] shared multiple risk factors in different domains (psychosocial, medical and socioeconomic risk factors). Multidimensional vulnerability was associated with adverse outcomes, such as premature birth and caesarean section.

**Conclusions:**

Co-existence of multiple risk factors in various domains is associated with adverse outcomes for mother and child. Early detection of vulnerability and strategies to improve parental health and well-being might benefit from focussing on different domains and combining medical and social care and support.

## Introduction

The first 1000 days of life, from preconception to the child’s second birthday, are crucial to children’s further physical, mental and social development. This critical and sensitive period is an important determinant of health and well-being in adulthood, as supported by the well-evidenced Developmental Origins of Health and Disease (DOHaD) concept.^1^^,^^2^ The DOHaD concept explains how experiences and exposures during early life, such as stress and nutrition, influence susceptibility to disease in later life and across generations, arguably through epigenetic mechanisms of foetal programming.^1^^,^^2^ Because of this intergenerational aspect, parents are the central focus to improve child health and advance health equity.^3^

To indicate subgroups of parents and their unborn or newborn children who are at higher risk of poor health or have lower access to healthcare, the concept of vulnerability is often used.^4–6^ Vulnerability reflects a complex and dynamic process. Simplified, various stressors at individual or contextual level (e.g. unemployment or living in a deprived neighbourhood) can act as risk factors to vulnerability, while protective factors (e.g. stable social network) might reduce or prevent vulnerability.^4^^,^^5^^,^^7^^,^^8^

Whether the presence of risk factors increases vulnerability and thereby hinder achieving one’s optimal health potential depends on the balance and interaction between risk and protective factors.^4^^,^^8^ While research on perinatal health has traditionally focussed on risk factors of a medical nature, there is now indisputable evidence for direct and indirect influences of social factors as well.^9–14^ The social, economic, cultural and environmental living conditions (i.e. social determinants of health) that shape parents’ and children’s daily experiences and thereby influence their health and development, are embedded in larger systems and structures, such as policies and laws.^3^^,^^15^

There is an international growing professional and political focus on early detection of vulnerability during the first 1000 days and development of effective strategies to improve parental health and well-being.^3^^,^^16^ For instance in the Netherlands, the government launched a nationwide ‘Solid Start’-programme in 2018 with the aim of providing each child the best start in life by strengthening collaboration between medical and social services, with a specific focus on families in vulnerable situations.^16^ Detecting vulnerability during pregnancy with the preventive purpose of countering suboptimal child health is challenging and can benefit from in-depth knowledge into vulnerability.

However, currently, little is known about the combination of different risk and protective factors to vulnerability and its influence on health outcomes. There seems to be few studies that consider protective factors to vulnerability and there is limited insight into clustering and underlying interactions, while it is recognized that especially the co-existence of risk factors can lead to adverse birth outcomes.^11^^,^^17^^,^^18^ Previous studies frequently explored the association between a limited number of predetermined, single risk factors and adverse birth outcome, but neglected co-existence of both protective- and risk factors that can influence outcomes.^12^^,^^18^^,^^19^

The aim of this study was to identify classes of vulnerability among pregnant women based on a wide range of social risk and protective factors in a latent class analysis (LCA). We conducted the LCA using Dutch observational nationwide data sources and self-reported data prior to pregnancy. In addition, we validated these classes by studying the association between latent class membership and various maternal and perinatal health outcomes and care utilization.

## Methods

### Data sources

This study utilized data from the nationwide population-based data-infrastructure DIAPER (acronym for Data-InfrAstructure for ParEnts and ChildRen). DIAPER integrates routinely collected observational data from three Dutch nationwide data sources (Perined, Vektis and Statistics Netherlands) at individual level. The Dutch Perinatal Registry ‘Perined’ collects routine care data on pregnancy after 22 weeks of gestation, birth and the first 28 days after birth, as supplied by midwives, gynaecologists and paediatricians.^20^ Healthcare information centre ‘Vektis’ collects claims data under the Dutch Healthcare Insurance Act and provides data on healthcare utilization and spending.^21^ ‘Statistics Netherlands’ collects and publishes data on societal matters and provides access to data through their System of Social Statistical Datasets (SSD).^22^^,^^23^ This linkable SSD-data covers nearly 20 themes, including health, welfare, income, education and labour.

We enriched DIAPER with self-reported data on health, well-being and lifestyle of the Public Health Monitor 2016 (PHM-2016).^24^ This is a health survey among a varying sample of the Dutch population aged 19 years and older, carried out every 4 years by the Community Health Services, Statistics Netherlands and the National Institute for Public Health and the Environment. The PHM-2016 had 457 153 participants and was mainly conducted from September–December 2016. Supplementary appendix S1 provides more information about the data sources.

### Study population

To ensure that information was not influenced by pregnancy itself, women were eligible for inclusion if these criteria were met: (i) they participated in the PHM-2016 (pre-pregnancy), (ii) they gave birth (livebirth or stillbirth) or had a termination of pregnancy before 1 January 2019 and (iii) pregnancy data in 2017 or 2018 were recorded within Perined. In case women had multiple pregnancies or births during the study period, only data on the first observation was included, to avoid duplication of women’s characteristics.

### Variables

The selection of variables for the LCA started with compiling a list of all possible risk and protective factors to vulnerability based on the framework of the National Academies of Sciences and Medicine,^3^ other scientific studies and definitions of vulnerability^4^^,^^5^^,^^8^ and expertise of the research team. Based on this list, 42 variables were available and selected in our data sources. These were divided into nine themes: individual characteristics, socioeconomic characteristics, lifestyle factors, household characteristics, self-reported health, healthcare expenditures and utilization, psychosocial characteristics, life-events and living conditions. The timing of the PHM-2016 was decisive in the choice for 1 October 2016 as baseline to include information. If data were available only on yearly basis, we included data from 2016. To increase interpretability, variables were categorized into two or three categories with the first category representing the risk factor to vulnerability. Supplementary appendix S1 provides a detailed overview of the variables, including definitions, categories and sources.

#### Outcomes

We studied the association between latent class membership and perinatal and maternal health outcomes and care utilization to validate classes. Perinatal health outcomes comprised: preterm birth (<37 weeks), small for gestational age (SGA, <10th percentile corrected for gestational age and foetal sex), preterm birth and/or SGA and admission to a neonatal intensive-care unit (NICU) after birth. Maternal health outcomes comprised: primary and secondary caesarean section, pre-eclampsia/hypertension and postpartum haemorrhage (≥1000 ml). Outcomes regarding healthcare utilization included: not having the first antenatal care appointment (i.e. booking visit) before the 10th week of pregnancy and not receiving postpartum care (at home) after birth. Supplementary appendix S1 provides more information.

### Statistical analyses

#### Latent class analysis

LCA is a data-driven analysis technique that aims to structure heterogeneity in a population by classifying individuals into unobserved—or latent—homogeneous classes.^25^ Structuring is based on included variables. Each class is denoted by conditional probabilities for each variable to take on a certain response value (e.g. 1 or 0), with the objective to categorize individuals into the smallest possible set of distinct and interpretable latent classes.

Using R version 3.6.2 (package poLCA), we estimated latent class models using all 42 variables with no prior assumptions about the optimal number of classes.^26^ Missing data were imputed through Multiple Imputation using Chained Equations (MICE) (Supplementary appendix S2). We started with a one-class model and stepwise increased to a 15-class model. Parameters of the latent class models were estimated by maximum likelihood. We considered both statistical fit as well as parsimony and interpretability to select the optimal model.^25^ To compare the competing models’ relative fit, we used the Akaike Information Criterion (AIC)^27^ and sample-size adjusted Bayesian Information Criterion (aBIC).^28^ Lower values indicate better fit of the model to the data. We also considered the fit-indices’ relative decrease, as done in previous studies,^29^ because a continuous decrease in the AIC is common with large sample sizes and the aBIC also may indicate towards a model with more classes than useful.^30^ We additionally reviewed the models’ entropy, which reflects how clearly the classes can be distinguished with scores ranging from 0 to 1 (optimum).^31^ We selected three preferred models based on their fit statistics and compared their item-response probabilities. The final model was selected based on parsimony and interpretability and women were classified into one of the identified classes based on predicted class membership (largest posterior probability). Further, to evaluate the LCA’s robustness, we performed two additional analyses. First, to unravel the impact of previous pregnancies, we excluded nullipara and conducted a LCA with additionally previous perinatal and pregnancy outcomes. Second, to evaluate whether similar vulnerability classes can be distinguished across women in the entire reproduction age, we repeated the LCA with a different study population consisting of all women between 19 and 44 years old.

#### Regression analysis

We studied the association between class membership and adverse outcomes by means of unadjusted logistic regression analysis. Results are reported as odds ratios (ORs) with 95% confidence interval (CI). A *p*-value of <0.05 was considered statistically significant.

## Results

The study population consisted of 4172 women, of whom 1129 had missing data (table 1). A five-class model was considered best (see Supplementary appendix S3 for fit-indices). The aBIC reached a minimum in the 12-class model, but did not show considerable improvement after models beyond seven classes when reviewing the relative fit (elbow shape). The AIC continuously decreased as expected. Entropy values were regarded best for models with two to five classes. We compared the interpretation of models with four, five and six classes and chose the five-class model for its interpretative and distinctive classes.

**Table 1:** Characteristics of the study population (including missing data)

		*n* (%)
Individual characteristics		
Age	19–23	306 (7.3)
	24–35	3528 (84.6)
	>35	338 (8.1)
Ethnicity	Non-Western	420 (10.1)
	Western	343 (8.2)
	Native Dutch	3409 (81.7)
Parity^a^	Nullipara	1755 (42.1)
	Primipara, multipara	2410 (57.8)
	*Missing*	*<10 (<0.2)*
Asylum seeker status	Yes	39 (0.9)
	No	4133 (99.1)
Socioeconomic characteristics		
Educational level	Low	328 (7.9)
	Moderate	1513 (36.3)
	High	2303 (55.2)
	*Missing*	*28 (0.7)*
Household income	Low	202 (4.8)
	Moderate	3348 (80.2)
	High	*591 (14.2)*
	*Missing*	*31 (0.7)*
Socioeconomic position	No income/receiving benefits	532 (12.8)
	Student	82 (2.0)
	Paid work	3502 (83.9)
	*Missing*	*56 (1.3)*
Debts and payment arrears	Yes	45 (1.1)
	No	4127 (98.9)
Insufficient financial resources	Yes	524 (12.6)
	No	3267 (78.3)
	*Missing*	*381 (9.1)*
Permanent contract	No	1929 (46.2)
	Yes	2243 (53.8)
Full-time contract	No	1925 (46.1)
	Yes	2247 (53.9)
Lifestyle factors		
Smoking	Yes	661 (15.8)
	No	3315 (79.5)
	*Missing*	*196 (4.7)*
Alcohol use	Yes (excessive)	418 (10.0)
	No	*3503 (84.0)*
	*Missing*	*251 (6.0)*
Physical activity	Less than recommended	1696 (40.7)
	As recommended or more	2158 (51.7)
	*Missing*	*318 (7.6)*
Body mass index (BMI)	Unhealthy BMI	1386 (33.2)
	Healthy BMI	2641 (63.3)
	*Missing*	*145 (3.5)*
Household characteristics		
Type of household	One-person/parent household	353 (8.5)
	Other	*3819 (91.5)*
Marital status	Unmarried	2147 (51.5)
	Married	2025 (48.5)
Dissolution of marriage	Yes	58 (1.4)
	No	4114 (98.6)
Household size	≥6 persons	93 (2.2)
	<6 persons	4079 (97.8)
Youth support uptake	Yes	102 (2.4)
	No	4070 (97.6)
Self-reported health		
Perceived health status	Negative	*465 (11.1)*
	Positive	3653 (87.6)
	*Missing*	*54 (1.3)*
Long-term illness	Yes	747 (17.9)
	No	3362 (80.6)
	*Missing*	*63 (1.5)*
Restricted by health	Yes	724 (17.4)
	No	3330 (79.8)
	*Missing*	*118 (2.8)*
Healthcare expenditures and utilization		
Overall healthcare expenditures	High	*824 (19.8)*
	Low-average	3297 (79.0)
	*Missing*	*51 (1.2)*
General practitioners’ (GP) expenditures	High	827 (19.8)
	Low-average	3308 (79.3)
	*Missing*	*37 (0.9)*
Hospital expenditures	High	413 (9.9)
	Low or none	3708 (88.9)
	*Missing*	*51 (1.2)*
Medication use	High	428 (10.3)
	Low or none	*3744 (89.7)*
Addiction related care uptake	Yes	23 (0.6)
	No	4149 (99.4)
Psychosocial characteristics		
Mental healthcare uptake	Yes	228 (5.5)
	No	3907 (93.6)
	*Missing*	*37 (0.9)*
Risk of depression or anxiety disorders	Moderate–high risk	1716 (41.1)
	No or low risk	2256 (54.1)
	*Missing*	*200 (4.8)*
Loneliness	Feeling lonely	*1100 (26.4)*
	Not feeling lonely	2719 (65.2)
	*Missing*	*353 (8.5)*
Feelings of control over life	Low	144 (3.5)
	Moderate	2741 (65.7)
	High	1006 (24.1)
	*Missing*	*281 (6.7)*
Mild intellectual disability	Yes	13 (0.3)
	No	4159 (99.7)
Life-events		
Crime suspect	Yes	*95 (2.3)*
	No	4077 (97.7)
Crime victim	Yes	874 (20.9)
	No	3298 (79.1)
Having been detained^a^	Yes	Not shown
	No	Not shown
History of frequent moving	Yes	1250 (30.0)
	No	2900 (69.5)
	*Missing*	*22 (0.5)*
Loss of a family member	Yes	147 (3.5)
	No	*4025 (96.5)*
Living conditions		
Home ownership	Rented	990 (23.7)
	Owner occupied	3099 (74.3)
	*Missing*	*83 (2.0)*
Motorized vehicle ownership	No	494 (11.8)
	Yes	3678 (88.2)
Proximity to general practitioners’ (GP) office	>3 km	265 (6.4)
	<3 km	3847 (92.2)
	*Missing*	*60 (1.4)*
Liveability neighbourhood	Low-mediocre	*273 (6.5)*
	High	3695 (88.6)
	*Missing*	*204 (4.9)*
Outcomes		
Preterm birth	Yes	277 (6.6)
	No	3895 (93.4)
Small for gestational age (SGA)	Yes	324 (7.8)
	No	3814 (91.4)
	*Missing*	*25 (0.6)*
Preterm birth and/or SGA	Yes	557 (13.4)
	No	3590 (86.0)
Admission to neonatal intensive-care unit (NICU)	Yes	130 (3.1)
	No	4042 (96.9)
Primary caesarean section	Yes	318 (7.6)
	No	3854 (92.4)
Secondary caesarean section	Yes	303 (7.3)
	No	3869 (92.7)
Pre-eclampsia/hypertension	Yes	250 (6.0)
	No	3922 (94.0)
Postpartum haemorrhage	Yes	265 (6.4)
	No	3907 (93.6)
No postpartum care (at home)	No postpartum care	258 (6.2)
	Postpartum care	3914 (93.8)
No antenatal care before week 10	No antenatal care before week 10	563 (13.5)
	Antenatal care before week 10	3236 (77.6)
	*Missing*	*373 (8.9)*

a Following guidelines of Statistics Netherlands, the data of some variables were rounded (parity) or not shown (having been detained) to prevent disclosure of information about individuals.

b Detailed definitions of variables and categories are provided in Supplementary appendix S1.

Missing data are shown in italic.

**Figure 1:**
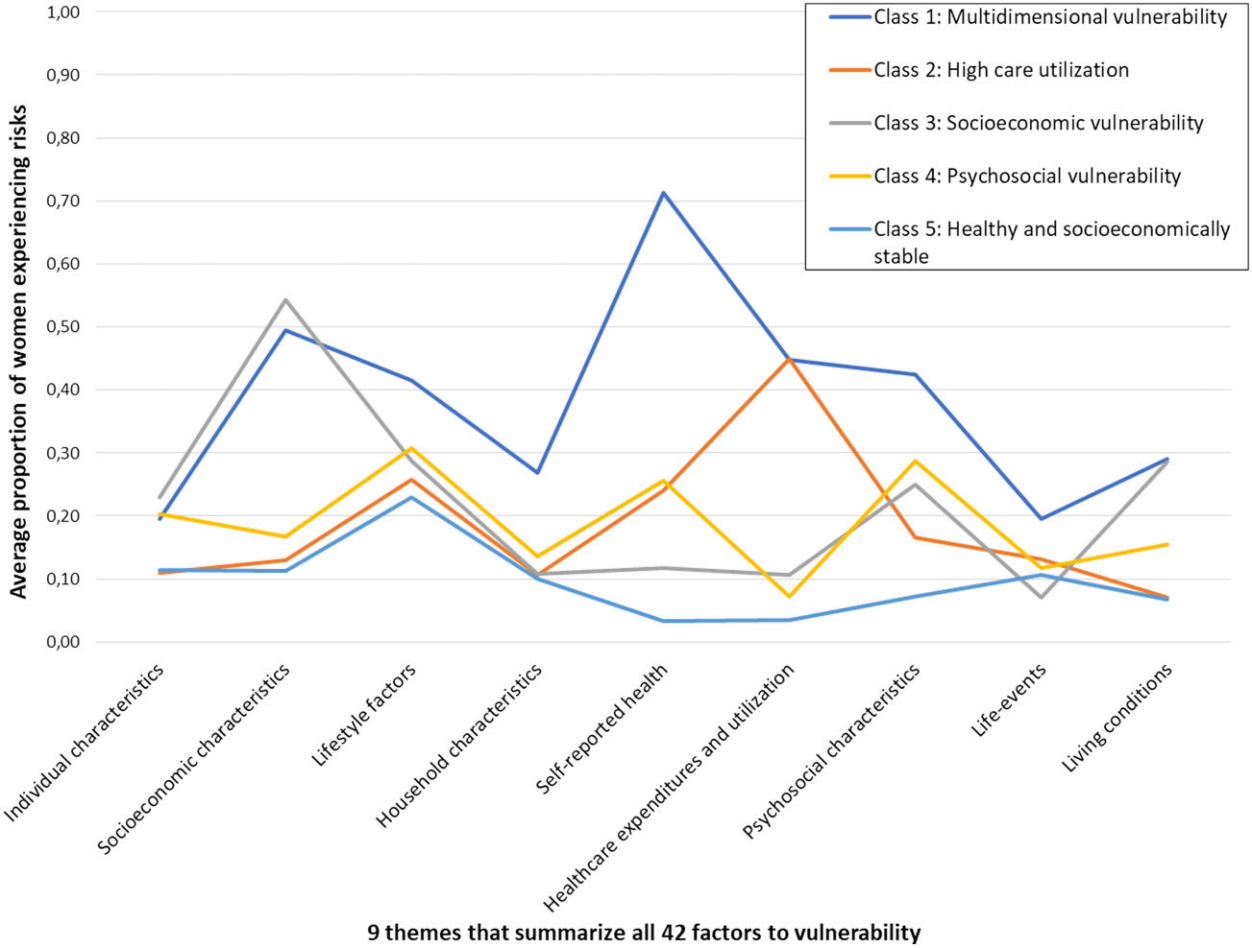
A visual representation of the five latent classes, described across the nine themes that summarize all 42 factors related to vulnerability. The vertical axis displays for each theme the average proportion of women within the categories that represent the risk factors (each first category in table 2). A higher score means that a higher proportion of women in a class have risk factors to vulnerability. An example: the theme ‘self-reported health’ consists of three factors: perceived health, long-term illness and restriction by health. For Class 1 (multidimensional vulnerability), the average proportion of women with a negative perceived health (0.7), long-term illness (0.68) and feelings of being restricted by health (0.76), is 0.71. This average proportion is displayed

The five-class model divided the study population into one class characterized by vulnerability in various domains, three classes characterized by vulnerability predominantly in one specific domain and one class with mainly protective factors (see table 2 for all class proportions and characteristics). Figure 1 provides a visual representation.

**Table 2:** Class proportions and descriptives of the final five-class model

	Class	1	2	3	4	5
	Label	Multidimensional vulnerability	High care utilization	Socioeconomic vulnerability	Psychosocial vulnerability	Healthy and socioeconomically stable
	Class proportions	0.06 (*n*=250)	0.11 (*n*=485)	0.09 (*n*=395)	0.24 (*n*=1005)	0.49 (*n*=2040)
Individual characteristics						
Age	19–23	0.14	0.03	*0.16*	0.13	0.03
	24–35	0.74	0.84	0.75	0.76	*0.92*
	>35	0.12	*0.13*	0.09	0.10	0.05
Ethnicity	Non-Western	0.26	0.02	*0.44*	0.13	0.02
	Western	0.06	0.08	*0.13*	0.11	0.06
	Native Dutch	0.68	0.90	0.43	0.76	*0.91*
Parity	Nullipara	0.38	0.39	0.23	*0.55*	0.41
	Primipara, multipara	0.60	0.61	*0.77*	0.45	0.59
Asylum seeker status	Yes	0.00	0.00	*0.09*	0.00	0.00
	No	0.98	*1.00*	0.91	*1.00*	*1.00*
Socioeconomic characteristics						
Educational level	Low	*0.30*	0.04	*0.30*	0.09	0.01
	Moderate	*0.54*	0.31	0.39	0.50	0.29
	High	0.16	0.65	0.30	0.41	*0.70*
Household income	Low	0.16	0.00	*0.30*	0.05	0.00
	Moderate	0.82	0.75	0.66	*0.90*	0.80
	High	0.00	*0.25*	0.03	0.05	0.20
Socioeconomic position	No income/receiving benefits	**0.62**	0.03	***0.87***	0.00	0.02
	Student	*0.06*	0.00	*0.06*	0.04	0.00
	Paid work	0.30	0.97	0.06	0.96	*0.98*
Debts and payment arrears	Yes	*0.12*	0.00	0.03	0.00	0.00
	No	0.88	*1.00*	0.97	*1.00*	*1.00*
Insufficient financial resources	Yes	***0***.***60***	0.07	0.35	0.27	0.02
	No	0.38	0.93	0.65	0.73	*0.98*
Permanent contract	No	**0.92**	0.32	***0.99***	0.45	0.34
	Yes	0.08	*0.68*	0.01	0.55	0.66
Full-time contract	No	**0.74**	0.45	***0.96***	0.31	0.40
	Yes	0.26	0.55	0.04	*0.69*	0.59
Lifestyle factors						
Smoking	Yes	*0.36*	0.16	0.14	0.25	0.12
	No	0.64	0.84	0.86	0.75	*0.88*
Alcohol use	Yes (excessive)	*0.14*	0.10	0.04	0.11	0.12
	No	0.86	0.90	*0.96*	0.89	0.88
Physical activity	Less than recommended	*0.52*	0.47	0.48	0.45	0.42
	As recommended or more	0.48	0.54	0.52	0.55	*0.58*
Body mass index (BMI)	Unhealthy BMI	***0.64***	0.30	0.49	0.42	0.26
	Healthy BMI	0.36	0.70	0.51	0.58	*0.74*
Household characteristics						
Type of household	One-person/parent household	*0.38*	0.03	0.10	0.15	0.03
	Other	0.62	*0.97*	0.90	0.85	*0*.*97*
Marital status	Unmarried	***0.66***	0.45	0.30	0.47	0.46
	Married	0.34	0.55	*0.70*	0.42	0.54
Dissolution of marriage	Yes	*0.08*	0.02	0.00	0.02	0.00
	No	0.92	0.99	*1.00*	0.98	*1.00*
Household size	≥6 persons	0.04	0.02	*0*.*10*	0.01	0.01
	<6 persons	0.96	0.98	0.90	0.98	*0*.*99*
Youth support uptake	Yes	*0.18*	0.01	0.04	0.03	0.00
	No	0.80	0.99	0.96	0.97	*1.00*
Self-reported health						
Perceived health status	Negative	***0.70***	0.12	0.10	0.20	0.00
	Positive	0.30	0.88	0.90	0.80	*1.00*
Long-term illness	Yes	***0.68***	0.32	0.09	0.28	0.06
	No	0.32	0.68	0.91	0.72	*0*.*94*
Restricted by health	Yes	***0.76***	0.28	0.16	0.29	0.04
	No	0.24	0.72	0.84	0.71	*0*.*96*
Healthcare expenditures and utilization						
Overall healthcare expenditures	High	**0.66**	***1.00***	0.16	0.05	0.04
	Low-average	0.34	0.00	0.84	0.95	*0.96*
General practitioners’ (GP) expenditures	High	***0.68***	0.33	0.23	0.21	0.10
	Low-average	0.30	0.67	0.77	0.79	*0.90*
Hospital expenditures	High	0.30	***0.69***	0.08	0.00	0.00
	Low or none	0.70	0.31	0.92	*1.00*	*1.00*
Medication use	High	*0.54*	0.23	0.06	0.10	0.03
	Low or none	0.46	0.77	0.94	0.90	*0.97*
Addiction related care uptake	Yes	*0.06*	0.00	0.00	0.00	0.00
	No	0.94	*1.00*	*1.00*	0.99	*1.00*
Psychosocial characteristics						
Mental healthcare uptake	Yes	*0.32*	0.12	0.01	0.06	0.01
	No	0.68	0.88	*0*.*99*	0.94	*0*.*99*
Risk of depression or anxiety disorders	Moderate—high risk	***0.86***	0.46	0.56	**0.71**	0.21
	No or low risk	0.12	0.54	0.44	0.28	*0*.*79*
Loneliness	Feeling lonely	***0.68***	0.22	0.56	0.57	0.14
	Not feeling lonely	0.32	0.78	0.44	0.43	*0*.*86*
Feelings of control over life	Low	*0.24*	0.03	0.11	0.10	0.00
	Moderate	0.72	0.75	0.76	*0*.*81*	0.63
	High	0.02	0.22	0.13	0.09	*0*.*37*
Mild intellectual disability	Yes	*0.02*	0.00	0.01	0.00	0.00
	No	0.98	*1.00*	0.99	*1.00*	*1.00*
Life-events						
Crime suspect	Yes	*0*.*14*	0.01	0.03	0.03	0.01
	No	0.86	*0*.*99*	0.97	0.97	*0*.*99*
Crime victim	Yes	*0*.*34*	0.24	0.11	0.23	0.20
	No	0.66	0.77	*0*.*89*	0.77	0.80
Having been detained	Yes	*0*.*02*	0.00	0.00	0.00	0.00
	No	0.98	*1.00*	*1.00*	*1.00*	*1.00*
History of frequent moving	Yes	*0*.*42*	0.36	0.20	0.30	0.29
	No	0.56	0.64	*0*.*80*	0.70	0.71
Loss of a family member	Yes	*0*.*06*	0.05	0.01	0.03	0.03
	No	0.92	0.96	*0*.*99*	0.97	0.97
Living conditions						
Home ownership	Rented	***0.64***	0.10	0.58	0.36	0.10
	Owner occupied	0.34	*0*.*90*	0.42	0.64	*0*.*90*
Motorized vehicle ownership	No	*0.32*	0.07	0.29	0.13	0.06
	Yes	0.66	0.93	0.71	0.87	*0*.*94*
Proximity to general practitioners’ (GP) office	>3 km	0.02	*0*.*08*	0.05	0.04	*0*.*08*
	<3 km	*0*.*98*	0.92	0.95	0.96	0.92
Liveability neighbourhood	Low-mediocre	0.18	0.03	*0*.*22*	0.09	0.03
	High	0.82	*0*.*97*	0.78	0.91	*0*.*97*

Proportions of risk factors (first category) >0.6 are shown in bold to indicate the higher occurrence of certain risk factors per class.

For each category, the class with the highest proportion is shown in italic.

Totals may not add up to 1.0 because of rounding. Following guidelines of Statistics Netherlands, the observed numbers in each category were rounded to five before calculating proportions in order to prevent the disclosure of information about individuals.

Class 1 (*n* = 250; 6.0%), was characterized by high proportions of almost all risk factors to vulnerability. Women in this class were likely to receive social benefits or to have no income (proportion of 0.62) and to live in a rented house (0.65). Related to health, Class 1 was characterized by high GP healthcare expenditures (0.67), long-term illness (0.68) and negative perceptions of health (0.70). These women had a high probability of feeling lonely (0.87) and a moderate to high risk of depression or anxiety (0.87). Considering the vulnerabilities in different areas (including psychosocial, medical and socioeconomic risk factors), Class 1 was named ‘multidimensional vulnerability’.

Class 2 (*n* = 485; 11.6%) was characterized by high healthcare expenditures. All women classified in this class had total healthcare expenditures in the highest quintile. Also, they frequently experienced high hospital care expenditures (0.69). Simultaneously, women in this class were likely to have protective factors including a healthy BMI (0.68), positive perception of health (0.87), high educational level (0.65), paid work (0.96), low probability of feeling lonely (0.78) and an owner-occupied house (0.90). Based on the dominant features, Class 2 was named ‘high care utilization’.

Class 3 (*n* = 395; 9.5%) was characterized in particular by high proportions of socioeconomic risk factors. Women in this class were likely to receive social benefits or have no income prior to pregnancy (0.87). They frequently lived in a rented house (0.58), had a non-Dutch background (0.56) and a low (0.30) or moderate (0.39) educational level. The probability of living in a neighbourhood with a low liveability score was highest in this class (0.22). When considering protective factors, these women were often married (0.70), had a positive perception of health (0.90) and low healthcare expenditures (0.83). Class 3 was named ‘socioeconomic vulnerability’.

Class 4 (*n* = 1005; 24%) was characterized by psychosocial health issues. The majority had a moderate to high risk of depression or anxiety disorders prior to pregnancy (0.71). These women were likely to feel lonely (0.57) and nullipara were overrepresented (0.55). Regarding protective factors, the majority had a full-time contract (0.69), an owner-occupied house (0.64) and no high healthcare expenditures (0.95). Class 4 was named ‘psychosocial vulnerability’.

Class 5 (*n* = 2040; 48.9%) was characterized by women with low probabilities of all risk factors to vulnerability before pregnancy. Instead, in general, these women had a positively perceived health (1.00), did not feel lonely (0.86), had a high educational level (0.70) and paid work (0.98). Women in Class 5 had the highest probability to experience high control over life (0.37). Class 5 was named ‘healthy and socioeconomically stable’.

The analyses in the two additional study populations (women who gave birth before and all women aged 19–44 years) showed similar results. The five-class model was preferred and classes could be interpreted similarly.

**Figure 2:**
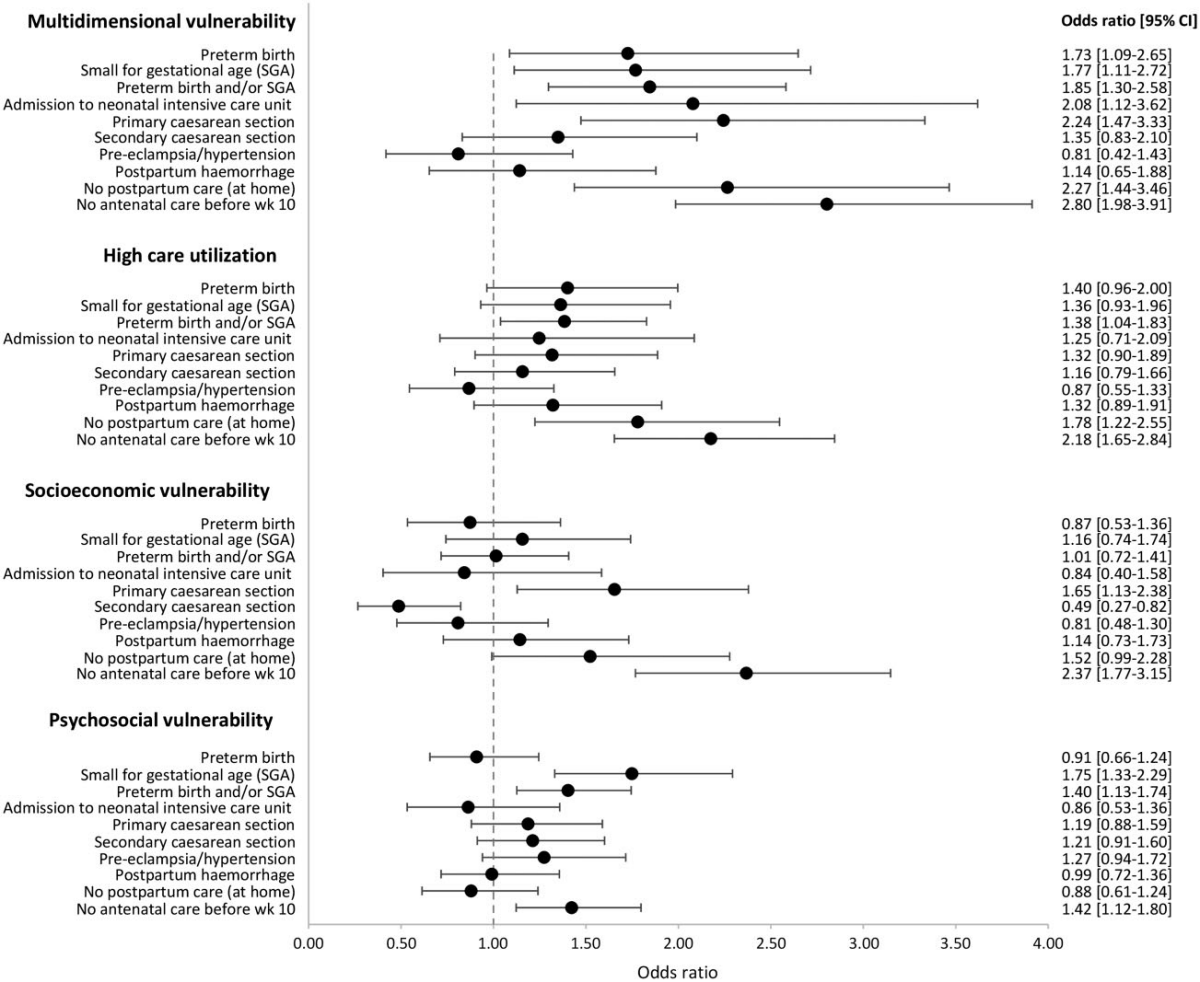
Likelihood of adverse perinatal and maternal health outcomes and healthcare utilization for four classes, compared to the reference-category ‘healthy and socioeconomically stable’. The figure shows the OR and 95% CI (graphically and in text)

Figure 2 shows associations between classes and adverse outcomes. Class 5 (healthy and socioeconomically stable) was the reference-category. Women classified in Class 1 (multidimensional vulnerability) were more likely to have babies who were born prematurely, SGA or admitted to a NICU. These women were also more likely to have a caesarean section. There were no significant associations found for other maternal health outcomes including hypertension/pre-eclampsia and postpartum haemorrhage. Compared to Class 5 (healthy and socioeconomical stable), all other classes except Class 4 (psychosocial vulnerability) were more likely to not receive postpartum care (at home) and to not receive antenatal care on time. Adverse outcomes were quite similar in Class 2 (socioeconomic vulnerability) and Class 5 (healthy and socioeconomically stable), except from the odds of planned caesarean section. Supplementary appendix S4 shows prevalences of outcomes for each class.

## Discussion

This study aimed to identify classes of vulnerability among pregnant women and to validate these classes by studying the association with adverse perinatal and maternal health outcomes and care utilization. The LCA procedure identified five classes with different combinations of risk and protective factors to vulnerability. Most women were classified into the ‘healthy and socioeconomically stable’-class with mainly protective factors. Women classified in the classes ‘high care utilization’, ‘socioeconomic vulnerability’ or ‘psychosocial vulnerability’ shared risk factors to vulnerability in one specific domain and protective factors in others. Women classified into the ‘multidimensional vulnerability’-class shared multiple risk factors in several domains (e.g. psychosocial, medical and socioeconomic) and were more likely to develop poor health outcomes, such as premature birth, SGA, caesarean section and NICU admission.

Our study showed that multidimensional vulnerability leads to experiencing worse outcomes compared to vulnerability on a single domain or no vulnerabilities. This indicates the importance of co-existence or clustering of multiple risk factors (such as no income, high healthcare expenditures and feelings of loneliness) in increasing the probability of adverse outcomes for mother and child. Our findings strengthen results from previous studies that aimed to explain differences in adverse outcomes by interrelated individual or contextual risk factors.^10^^,^^11^^,^^17^ Previous LCA studies also led to classes of pregnant women with different health behaviours, psychosocial or socioeconomic characteristics that show differences in outcomes, although these studies included less factors and domains, and other populations in comparison to our study.^17^^,^^32^^,^^33^ The findings do not inform us on how risk factors interplay and lead to adverse health outcomes. The syndemic model provides a perspective on this interplay by describing how co-occurring health adversities are fuelled by different social and contextual factors that interact and increase the health burden of both mental and physical illness.^34^ This suggests the need to combine social and medical care and support, instead of focussing on the separate domains to combat multidimensional vulnerability.

We found that women with socioeconomic vulnerability generally did not experience worse outcomes. This finding is not in congruence with previous research indicating that adverse perinatal health outcomes are more prevalent among women with a low socioeconomic status (SES).^9^^,^^10^^,^^14^ Previous studies often focussed on a limited number of risk factors or domains, or used more traditional (regression) techniques to study the relation between SES and outcomes. However, as the impact of risk factors can depend on other factors, it is important to step away from traditional independent ‘ceteris paribus’ linear effect assumption of social determinants. Therefore, we used LCA as analytical approach that considers the combination of both risk and protective factors, allowing a more comprehensive approach to study vulnerability. Protective factors (e.g. social support) can act as positive exposures or buffering mechanisms that promote resilience and improve health.^3^^,^^8^^,^^35^^,^^36^ This indicates the importance of acknowledging both strengths and challenges in families to create a supportive environment for early development.^37^ Additionally, low SES may not necessarily be a risk factor for adverse outcomes unless it coincides with other hardships. The relation between SES and health can be described by processes such as social causation (adverse conditions of poverty impact health through, for example, stress and food insecurity) and health selection (people with worse physical or mental health outcomes fall into poverty through, for example, stigma, health expenditures and lower productivity).^38^ This increases the importance for healthcare professionals to understand different domains of vulnerability and tailor the need for support to the individual.^39^^,^^40^

Our findings reveal a difference in care utilization patterns. The ‘healthy and socioeconomically stable’-class was most likely to receive early antenatal care and postpartum care (at home). This corresponds to findings of Grabovschi *et al*.^6^ in their scoping review into vulnerability. People with higher vulnerability levels (i.e. multiple vulnerability aspects) have higher healthcare needs, but less access to services and lower quality of healthcare. This raises questions about whether current support meets parents’ needs.

The main strength of this study is that we linked routinely collected nationwide observational data sources to self-reported data on health, well-being and lifestyle. This offered the opportunity to include data on a wide range of medical and social factors for a large group of pregnant women to better understand vulnerability. While previous studies often had a unidimensional perspective to vulnerability (focussing on single risk factors such as individual SES, or neighbourhood SES on aggregated level), we could unravel the difference between unidimensional and multidimensional types of vulnerability due to our extensive dataset. Another strength is that we included protective factors, while most studies focus primarily on factors that increase the risk of adverse outcomes and less on protective factors that might counteract these effects.^18^^,^^19^ Unfortunately, data on topics such as nutrition, stress, health literacy, preconception care and adverse childhood experiences were not available, while these factors could provide additional insights into vulnerability. Next, using largest posterior probability to assign women to classes is a limitation, because not all women are fully representative of one class only. Our study was moreover limited by not including the father or woman’s partner, despite growing evidence of their importance in promoting healthy pregnancy, childbirth and child-outcomes. Another limitation relates to the representativeness of the study population due to using the PHM-2016. Compared to all other pregnant women in 2017/2018, women in our study less often had a low income (5% vs. 8%), low educational level (8% vs. 12%) and migration background (18% vs. 32%). Since generally people with higher vulnerability less often participate in research, we assume that the size of the multidimensional vulnerability-class is an underestimation. Nevertheless, since we could identify classes of vulnerability and differentiate between single and multidimensional vulnerability, we expect that their characteristics are also applicable beyond the study population. Similar results from our additional analyses strengthen this expectation. Nevertheless, our approach and findings should be validated in other cohorts and countries and until then be interpreted with caution.

Our findings can have several implications for practice and research. We believe that screening instruments for vulnerability before and during pregnancy could benefit from including a balanced set of both risk and protective factors. In refining screening instruments, we have to consider the various criteria for responsible screening, such as the availability of associated care or support strategies.^41^ Greater consciousness among healthcare providers regarding the complexity of vulnerability in terms of risk and protective factors and personal perceptions could enhance the provision of person-centred care and support.^6^^,^^40^^,^^42^ Multiple studies argue that future strategies should also pay attention to underlying, root causes of vulnerability in policies, laws and governance.^3^^,^^15^^,^^43^ Advancing health equity requires both individual-level interventions targeted at vulnerable individuals as well as systemic-level change.^3^^,^^15^^,^^43^ Factors related to housing, education and social security for example, frequently lie upstream of individual lifestyle and behavioural factors modifiable through individual-level interventions. Findings of our study can be input for longitudinal monitoring of vulnerability at population level. Future research is needed to identify if vulnerability classes can be identified using solely routinely collected population data, without using self-reported data. Additionally, more research is necessary regarding the role of the father or woman’s partner in relation to vulnerability.

In conclusion, there is growing attention for early detection of vulnerability and implementing effective strategies to improve health and well-being of current and next generations. Results of this data-driven study suggest that several vulnerability classes can be distinguished among pregnant women in the Netherlands. The co-existence of risk factors in multiple domains leads to more adverse outcomes for mother and child. Effective strategies, starting preconceptionally, should include both medical and social care and support.

## Supplementary data

Supplementary data are available at *EURPUB* online.

## Supplementary Material

ckac170_Supplementary_Data

## Data Availability

We are unable to share the individual data used for this study as data linkage and analysis was conducted within the highly safeguarded Remote Access (RA) platform of Statistics Netherlands.^23^ All data within this platform are pseudonymized to ensure data safety and confidentiality. Access to the data from Perined, Vektis, Statistics Netherlands and the Public Health Monitor 2016 can be requested from the relevant parties. Previous evidence supports the influence of social factors on maternal and perinatal health, but few studies consider the combination of different social risk and protective factors to vulnerability. Pre-pregnancy data of 4172 women on a wide range of social risk and protective factors to vulnerability were used to identify latent vulnerability classes. Five classes could be distinguished: multidimensional vulnerability, high care utilization, socioeconomic vulnerability, psychosocial vulnerability and a healthy and socioeconomically stable-class. Multidimensional vulnerability, characterized by experiencing risk factors in different domains and few protective factors, was associated with adverse outcomes for mother and child, while experiencing risk factors solely in one domain was not necessarily associated with adverse outcomes. Public health programmes should start preconceptionally, include both medical and social care and support, and be attentive to systemic causes of vulnerability.

## Notes

41 References 41–43 are provided in the Supplementary References.
