# Supplementary material for: Defining vulnerability subgroups among pregnant women using pre-pregnancy information: a latent class analysis
Source: Eur J Public Health. 2022 Dec 14;33(1):25–34. doi: 10.1093/eurpub/ckac170 (PMC10263266; doi:10.1093/eurpub/ckac170)
Supplement: ckac170_Supplementary_Data [file ckac170_supplementary_data.zip › ckac170_Supplementary_Data/ejph-2022-06-om-0336-File006.docx]

Appendix 1. Description of data sources, variables and outcomes included

**Data sources**

The Dutch Perinatal Registry Perined collects routine care data on pregnancy after 22 weeks of gestation, birth and the first 28 days after birth, as supplied by midwives, gynecologists and pediatricians.^[[1]](#footnote-2)^ Perined includes data on type of care used and health outcomes of mother and child. From 2000 onwards, Perined contains data on 96 – 99% of all births.

Healthcare information center Vektis collects claims data under the Dutch Healthcare Insurance Act and provides data on healthcare utilisation and spending.^[[2]](#footnote-3)^ Data on 99% of the Dutch (insured) population is available. DIAPER contains detailed claims data of Vektis as available from 2015.

Statistics Netherlands collects and publishes data on societal matters and provides access to data through their System of Social Statistical Datasets (SSD).^[[3]](#footnote-4),^^[[4]](#footnote-5)^ This linkable SSD-data covers nearly 20 themes, including health, welfare, income, education and labour. Data originate from various (governmental) organisations, including municipalities and the National Tax Authority.

We enriched DIAPER with self-reported data on health, well-being and lifestyle of the Public Health Monitor 2016 (PHM-2016). This is a health survey among a varying sample of the Dutch population aged 19 years and older, carried out every four years by the Community Health Services (GGD), Statistics Netherlands and the National Institute for Public Health and the Environment. The PHM-2016 had 457.153 participants and was mainly conducted from September-December 2016.

**Variables in LCA**

| **Variable** | **Definition** | **Categories** | **Data source*** |
| --- | --- | --- | --- |
| **Individual characteristics** |  |  |  |
| Age | Age of the woman in categories, based on an increased risk for pregnancy complications related to age ^[[5]](#footnote-6),^^[[6]](#footnote-7)^ | 19– 23; 24 – 35; >35 | SSD |
| Ethnicity | Migration status based on birthplace of parents, following categorization of Statistics Netherlands^[[7]](#footnote-8)^ A person with a migration background is a person of whom at least one parent was born abroad. | Non-western; Western; native Dutch | SSD |
| Parity | The number of times that a woman has given birth after a gestational period of 24 weeks or more: 0 times (nullipara), 1 time (primipara), 2 or more times (multipara) | Nullipara; primipara or multipara | Perined |
| Asylum seeker status | Registered as asylum seeker, status holder or refugee and included in the municipal population register. | Yes; no | SSD |
| **Socioeconomic characteristics** |  |  |  |
| Educational level | Highest completed education, according to the International Standard Classification of Education and following categorization of Statistics Netherlands^[[8]](#footnote-9)^ | Low; moderate; high | PHM-2016 and SSD |
| Household income | Household income based on percentage groups of standardized disposable income of private households; low is <10^th^ percentile, moderate is 10^th^ – 90^th^ percentile and high is > 90^th^ percentile | Low; moderate; high | SSD |
| Socioeconomic position | Source of income, if any | No income or receiving benefits; student; paid work | SSD |
| Debts or payment arrears | Difficulty paying bills, based on registration of debt restructuring and/or delayed health insurance payments for more than six months | Yes; no | SSD |
| Insufficient financial resources** | Having insufficient means, based on the question: ‘in the past 12 months, have you struggled to make ends meet on your household income?’ | Yes; no | PHM-2016 |
| Permanent contract | Having a permanent type of work contract | No; yes | SSD |
| Fulltime employment | Being fulltime employed | No; yes | SSD |
| **Lifestyle factors** |  |  |  |
| Smoking** | Based on the question: ‘do you ever smoke?’ | Yes; no | PHM-2016 |
| Alcohol use | Alcohol use, categorized as excessive based on the number of glasses of alcohol in a week (> 7), following Dutch ’Guidelines for Good Nutrition’^[[9]](#footnote-10)^ | Yes (excessive); no | PHM-2016 |
| Physical activity | Compliance with guidelines for physical activity (> 150 minutes of moderate to vigorous intensive activities every week, as well as muscle and bone strengthening activities) as measured by the SQUASH-questionnaire^[[10]](#footnote-11)^. | Less than recommended; as recommended or more | PHM-2016 |
| Body Mass Index (BMI) | BMI based on height and weight. According to the International Obesity Task Force, BMI < 15 is underweight, BMI 18,5 – 25 is normal weight and BMI ≥25 overweight^[[11]](#footnote-12)^. Underweight and overweight are categorized into ‘unhealthy BMI’. Normal weight is categorized into ‘healthy BMI’ | Unhealthy BMI; healthy BMI | PHM-2016 |
| **Household characteristics** |  |  |  |
| Type of household | Type of household was categorized based on registrations of persons living at the same home address. Women without a registered partner were classified as a one-person household (without children) or a one-parent household (with children). All other types were categorized into ‘other’ | One-person or one-parent household; other | SSD |
| Marital status | Marital status of the woman | Unmarried; married | SSD |
| Dissolution of marriage*** | Having been divorced [2012-2016] | Yes; no | SSD |
| Household size | Number of persons registered at the same home address | ≥6 persons; <6 persons | SSD |
| Youth support*** | Previously received youth support for children within the family [2015-2016] | Yes; no | SSD |
| **Self-reported health** |  |  |  |
| Perceived health status | Based on the question: ‘how is your health in general?’ and categorized into negative (very bad or bad) and positive (very good, good or moderate) | Negative; positive | PHM-2016 |
| Long-term illness | Based on the question: ‘do you have one or more long-term (≥ 6 months) illnesses or disorders?’ | Yes; no | PHM-2016 |
| Restricted by health | Based on the question ‘to what extent have you been limited in activities due to health for the last 6 months or longer?’ into yes (severely limited or limited) and no (not limited). | Yes; no | PHM-2016 |
| **Healthcare expenditures and utilization** |  |  |  |
| Total healthcare expenditures | Total healthcare expenditures in 2016 subdivided into quintiles and divided into high (quintile 5) and low-average (quintile 1 to 4). In case a woman was pregnant in 2016, total healthcare expenditures of the previous year (2015 or 2014) were included. | High; low-average | SSD (Vektis) |
| General practitioners’ (GP) expenditures | GP expenditures in 2016 subdivided into quintiles and divided into high (quintile 5) and low-average (quintile 1 to 4) | High; low-average | SSD (Vektis) |
| Hospital expenditures | Hospital expenditures in 2016, categorized using a cut-off value corresponding to the highest 10% of expenditures among all women with hospital expenditures. In case a woman was pregnant in 2016, hospital expenditures of the previous year (2015 or 2014) were included, to avoid including pregnancy related health care costs. | High; low or none | SSD (Vektis) |
| Medication use | Number of registered types of medication used in 2016 categorized into high (≥5 different types) and low (<5) according to the most common definition of polypharmacy^[[12]](#footnote-13)^ | High; low or none | SSD |
| Addiction related care uptake *** | The presence or history of a diagnosis-treatment combination for mental healthcare related to addiction [2011-2016] | Yes; no | SSD |
| **Psychosocial characteristics** |  |  |  |
| Mental healthcare uptake | Expenditures that were made regarding mental healthcare services | Yes; no | SSD (Vektis) |
| Risk of depression or anxiety disorders** | Based on ten questions of the Kessler Psychological Distress scale^[[13]](#footnote-14)^, categorized into moderate to high risk and no or low risk | Moderate-high risk; no or low risk | PHM-2016 |
| Loneliness** | Based on 11 statements of the De Jong – Gierveld scale^[[14]](#footnote-15)^, categorized into feeling lonely (moderate – severe) and not feeling lonely | Feeling lonely; not feeling lonely | PHM-2016 |
| Feelings of control over life** | Based on seven statements^[[15]](#footnote-16)^ of the Pearlin Mastery scale and categorized into low (insufficient), moderate and high | Low; moderate; high | PHM-2016 |
| Mild intellectual disability | Registered indication for a mild intellectual disability as recorded in data sources of occupational disabilities, sheltered employment and entitlement to the Long-Term care Act. | Yes; no | SSD |
| **Life-events** |  |  |  |
| Crime suspect******* | Having been registered as a crime suspect [2009-2016] | Yes; no | SSD |
| Crime victim*** | Having been a victim of a crime that was reported to the police [2011-2016] | Yes; no | SSD |
| Detention*** | Having been detained [2004-2016] | Yes; no | SSD |
| Frequent moving | A change of registered home address of more than five times in the past five years | Yes; no | SSD |
| Loss of a family member*** | Loss of a parent and/or child [2011-2016] | Yes; no | SSD |
| **Living conditions** |  |  |  |
| Home ownership | The home ownership of the woman’s home address | Rented; owner-occupied | SSD |
| Motorized vehicle ownership | A registered motorized vehicle at the woman’s home address, according to the Dutch vehicle registration authority | No; yes | SSD |
| Proximity to general practitioners’ (GP) office | Distance (by road) between registered home address and the nearest GP’s office | > 3 km; < 3 km | SSD |
| Liveability of the neighbourhood | According to the Dutch Leefbaarometer^[[16]](#footnote-17)^, based on various elements including housing characteristics, residents, distance to services, safety and physical environment. | Low-mediocre; high | SSD |

* SSD and Perined are nationwide observational data sources, the Public Health Monitor 2016 (PHM-2016) contains self-reported data.
** The data of the PHM-2016 is collected through a combination of the regular Health Survey of Statistics Netherlands (98% of the study population) and the health surveys of the Municipal Health Services (2% of the study population). The variables indicated by the asterisks were not included in the regular Health Survey of Statistics Netherlands.
*** The number of included years was based on data-availability, which varied per item.

**Outcomes**

| **Outcome** | **Definition** | **Categories** | **Datasource** |
| --- | --- | --- | --- |
| Preterm birth | Birth occurring from 24 weeks of gestation and before 37 weeks of gestation | Yes; no | Perined |
| Small for gestational age (SGA) | Birth weight below 10th percentile, corrected for gestational age and fetal sex^[[17]](#footnote-18)^ | Yes; no | Perined |
| Preterm birth and/or SGA | Baby born prematurely and/or SGA, following definitions above | Yes; no | Perined |
| Admission to neonatal intensive care unit (NICU) | Admission to the Neonatal Intensive Care Unit after birth | Yes; no | Perined |
| Primary caesarean section | Birth by caesarean section, decision taken before the start of birth | Yes; no | Perined |
| Secondary caesarean section | Birth by caesarean section, decision taken during birth | Yes; no | Perined |
| Pre-eclampsia/hypertension | Having pre-eclampsia or maternal hypertension (high blood pressure), chronic or pregnancy induced. | Yes; no | Perined |
| Postpartum haemorrhage | Heavy bleeding after birth (>1000 ml blood loss) | Yes; no | Perined |
| No postpartum care (at home) | Woman does not receive postpartum care at home by a maternity care assistant^[[18]](#footnote-19)^ | No postpartum care (at home); postpartum care (at home) | Vektis |
| No antenatal care before wk 10 | Not having the first antenatal care appointment (i.e., booking visit) before the 10th week of pregnancy | No antenatal care before wk 10; antenatal care before wk 10 | Perined |

1. Perined. Over Perined [about Perined] n.d. [Available from: https://www.perined.nl/over-perined]. [↑](#footnote-ref-2)
2. de Boo A. Vektis 'Informatiecentrum voor de zorg’ [Vektis 'Information center for healthcare']. Tijdschrift voor gezondheidswetenschappen. 2011;89(7):358-9 [↑](#footnote-ref-3)
3. Bakker BF, Van Rooijen J, Van Toor L. The system of social statistical datasets of Statistics Netherlands: An integral approach to the production of register-based social statistics. Statistical Journal of the IAOS. 2014;30(4):411-24. [↑](#footnote-ref-4)
4. Statistics Netherlands (CBS). About CBS. n.d. [Available from: https://www.cbs.nl/en-gb/about-us/organization] [↑](#footnote-ref-5)
5. ^5^ Londero, A.P., Rossetti, E., Pittini, C. et al. Maternal age and the risk of adverse pregnancy outcomes: a retrospective cohort study. BMC Pregnancy Childbirth 19, 261 (2019). https://doi.org/10.1186/s12884-019-2400-x. [↑](#footnote-ref-6)
6. FIOM. Jong ouderschap. Available from: https://fiom.nl/kenniscollectie/jong-ouderschap [↑](#footnote-ref-7)
7. Statistics Netherlands (CBS). Person with a migration background. Available from: https://www.cbs.nl/en-gb/our-services/methods/definitions/person-with-a-migration-background [↑](#footnote-ref-8)
8. Statistics Netherlands (CBS). Standaard onderwijsindeling 2016 (The Dutch standard classification of education). Available from: https://www.cbs.nl/nl-nl/onze-diensten/methoden/classificaties/onderwijs-en-beroepen/standaard-onderwijsindeling--soi--/standaard-onderwijsindeling-2016 [↑](#footnote-ref-9)
9. Gezondheidsraad [Dutch Health Council]. Richtlijnen goede voeding 2006 [Guidelines good nutrition 2006]. Den Haag: Gezondheidsraad 2006. [↑](#footnote-ref-10)
10. Gezondheidsraad [Dutch Health Council]. Beweegrichtlijnen 2017 [exercise guidelines 2017]. Den Haag: Gezondheidsraad, 2017. [↑](#footnote-ref-11)
11. Cole TJ, Bellizzi MC, Flegal KM, Dietz WH. Establishing a standard definition for child overweight and obesity worldwide: international survey. BMJ. 2000;320(7244):1240-3. [↑](#footnote-ref-12)
12. Masnoon N, Shakib S, Kalisch-Ellett L, Caughey GE. What is polypharmacy? A systematic review of definitions. BMC geriatrics. 2017;17(1):1-10. [↑](#footnote-ref-13)
13. Kessler RC, Andrews G, Colpe LJ, Hiripi E, Mroczek D, et al. Short screening scales to monitor population prevalences and trends in non-specific psychological distress. Psychological medicine. 2002;32(6):959. [↑](#footnote-ref-14)
14. De Jong Gierveld J, Van Tilburg T. Manual of the loneliness scale 1999. Department of Social Research Methodology, Vrije Universiteit Amsterdam, Amsterdam (updated version 1801 02). 1999. [↑](#footnote-ref-15)
15. Pearlin LI, Schooler C. The structure of coping. Journal of health and social behavior. 1978:2-21. [↑](#footnote-ref-16)
16. Leidelmeijer, K., Marlet, G., Ponds, R., Schulenberg, R., van Woerkens, C., & van Ham, M. V. M. Leefbaarometer 2.0: instrumentontwikkeling. Rigo Research en Advies & Atlas voor de gemeenten.2014 [↑](#footnote-ref-17)
17. Hoftiezer, L., Hof, M. H., Dijs-Elsinga, J., Hogeveen, M., Hukkelhoven, C. W., & van Lingen, R. A. From population reference to national standard: new and improved birthweight charts. American journal of obstetrics and gynecology. 2019; 220(4): 383. [↑](#footnote-ref-18)
18. Laureij, L. T., van der Hulst, M., Lagendijk, J., Been, J. V., Ernst-Smelt, H. E., Franx, A., & Lugtenberg, M. Insight into the process of postpartum care utilisation and in-home support among vulnerable women in the Netherlands: an in-depth qualitative exploration. BMJ open. 2021; 11(9): e046696. [↑](#footnote-ref-19)
