# Supplementary material for: Defining vulnerability subgroups among pregnant women using pre-pregnancy information: a latent class analysis
Source: Eur J Public Health. 2022 Dec 14;33(1):25–34. doi: 10.1093/eurpub/ckac170 (PMC10263266; doi:10.1093/eurpub/ckac170)
Supplement: ckac170_Supplementary_Data [file ckac170_supplementary_data.zip › ckac170_Supplementary_Data/ejph-2022-06-om-0336-File007.docx]

Appendix 2. Details on the multiple imputation process

The missing data (range missing values per variable: 0 to 9.1%) for the latent class analysis were imputed using multiple imputation^[[1]](#footnote-1)^. We created five imputed datasets in R through the package Multiple Imputation using Chained Equations (MICE)^[[2]](#footnote-2)^. Model development was done in each set and the outcomes were compared. The outcomes were highly similar: they let to the same conclusions for both fit-statistics and class-specific probabilities. Subsequent analyses and the presentation of results was done for one randomly chosen imputed dataset.

|  | Multiple Imputation |
| --- | --- |
| Software used | R version 3.6.2 |
| Imputation method and key settings | Fully conditional specification (package mice version 3.13.0)*;* maximum iterations: 5 |
| Number of imputed datasets created | 5 |
| Analyses variables | Age; Ethnicity; Parity; Asylum seeker status; Educational level; Household income; Socioeconomic position; Debts or payment arrears; Insufficient financial resources*; Permanent contract; Fulltime employment; Smoking*; Alcohol use; Physical activity; Body Mass Index (BMI); Type of household; Marital status; Dissolution of marriage; Household size; Youth support; Perceived health status; Long-term illness; Restricted by health; Total healthcare expenditures; General Practitioners’ expenditures; Hospital expenditures; Medication use; Addiction related care uptake; Risk of depression or anxiety disorders*; Loneliness*; Feelings of control over life*; Mental healthcare use; Mild intellectual disability; Crime suspect; Crime victim; Detention; Frequent moving; Loss of a family member ; Home ownership; Motorized vehicle ownership; Proximity to the GP’s office; Liveability of the neighbourhood |
| Auxiliary variables | 11 variables for loneliness (statements + sum scores); 9 variables for control over life (statements + sum scores); 11 variables for depression or anxiety disorders (statements + sum scores); 2 variables for alcohol use; language of questionnaire; 7 variables for noise nuisance; 2 variables for socioeconomic status; 3 variables for crime victim; 2 variables for addiction related care uptake; 2 variables for loss of family member; 2 variables for insufficient financial resources; number of movements; number of life-events. |
| Treatment of continuous data | Predictive mean matching |
| Treatment of binary data | Logistic regression |
| Treatment of unordered categorical data | Polytomous logistic regression |
| Population | For the imputation we used additional data from the Public Health Monitor 2016 and Statistics Netherlands (SSD).  There were 3043 complete cases and 1129 women with missing data. These women mostly had missing data on one variable (n = 641). |

* The data of the Public Health Monitor 2016 is collected through a combination of the regular Health Survey of Statistics Netherlands (98% of the study population) and the health surveys of the Municipal Health Services (2% of the study population). The variables indicated by the asterisks were not included in the regular Health Survey of Statistics Netherlands and thus contain missing values.

1. Sterne JA, White IR, Carlin JB, et al. Multiple imputation for missing data in epidemiological and clinical research: potential and pitfalls. Bmj. 2009;338:b2393. [↑](#footnote-ref-1)
2. Buuren van S, Groothuis-Oudhoorn K. mice: Multivariate Imputation by Chained Equations in R. Journal of Statistical Software. 2011;45:1-67. [↑](#footnote-ref-2)
