# Supplementary material for: Defining vulnerability subgroups among pregnant women using pre-pregnancy information: a latent class analysis
Source: Eur J Public Health. 2022 Dec 14;33(1):25–34. doi: 10.1093/eurpub/ckac170 (PMC10263266; doi:10.1093/eurpub/ckac170)
Supplement: ckac170_Supplementary_Data [file ckac170_supplementary_data.zip › ckac170_Supplementary_Data/ejph-2022-06-om-0336-File008.docx]

Appendix 3*.* Fit results of LCA model with increasing number of classes.

| **Model*** | **Akaike Information Criterion (AIC)** | **Relative decrease AIC** | **Size adjusted Bayesian Information Criterion (aBIC)** | **Relative decrease aBIC** | **Bayesian Information Criterion (BIC)** | **Relative decrease BIC** | **Entropy** |
| --- | --- | --- | --- | --- | --- | --- | --- |
| Model 1 | 144228·5 | *NA* | 144380·1 | *NA* | 144532·6 | *NA* | NA |
| Model 2 | 137476·2 | *-0·047* | 137782·5 | *-0·046* | 138090·8 | *-0·045* | 0·807 |
| Model 3 | 135647·8 | *-0·013* | 136108·9 | *-0·012* | 136572·8 | *-0·011* | 0·781 |
| Model 4 | 134443·5 | *-0·009* | 135059·5 | *-0·008* | 135679·1 | *-0·007* | 0·803 |
| Model 5 | 133396·3 | *-0·008* | 134167 | *-0·007* | 134942·3 | *-0·005* | 0·784 |
| Model 6 | 132491·5 | *-0·007* | 133417 | *-0·006* | 134348 | *-0·004* | 0·771 |
| Model 7 | 131736·2 | *-0·006* | 132816·5 | *-0·005* | 133903·2 | *-0·003* | 0·774 |
| Model 8 | 131271·7 | *-0·004* | 132817 | *-0·002* | 133749·1 | *-0·001* | 0·680 |
| Model 9 | 130858·8 | *-0·003* | 132506·7 | *-0·002* | 133646·7 | *-0·001* | 0·757 |
| Model 10 | 130494·3 | *-0·003* | 132248·6 | *-0·002* | 133592·7 | *0* | 0·645 |
| Model 11 | 130226·1 | *-0·002* | 132038·8 | *-0·002* | 133635 | *0* | 0·625 |
| Model 12 | 130006·5 | *-0·002* | 131925·4 | *0* | 133725·8 | *0·001* | 0·537 |
| Model 13 | 129830·9 | *-0·001* | 131860·6 | *0* | 133860·7 | *0·001* | 0·524 |
| Model 14 | 129699·4 | *-0·001* | 131863 | *0* | 134039·7 | *0·001* | 0·531 |
| Model 15 | 129599·9 | *-0·001* | 131918·3 | *0* | 134250·7 | *0·002* | NA |

*The model number reflects the number of classes included in each model. For example, model 5 reflects a model with 5 classes.
