# Supplementary material for: Defining vulnerability subgroups among pregnant women using pre-pregnancy information: a latent class analysis
Source: Eur J Public Health. 2022 Dec 14;33(1):25–34. doi: 10.1093/eurpub/ckac170 (PMC10263266; doi:10.1093/eurpub/ckac170)
Supplement: ckac170_Supplementary_Data [file ckac170_supplementary_data.zip › ckac170_Supplementary_Data/ejph-2022-06-om-0336-File009.docx]

Appendix 4*.* Prevalence of outcomes for the five latent classes

|  | **Multidimensional vulnerability** | **High care utilization** | **Socioeconomic vulnerability** | **Psychosocial vulnerability** | **Healthy and socioeconomically stable** |
| --- | --- | --- | --- | --- | --- |
| Preterm birth | 10.4% | 8.6% | 5.6% | 5.8% | 6.3% |
| Small for gestational age (SGA) | 10.5% | 8.3% | 7.1% | 10.4% | 6.2% |
| Preterm birth and/or SGA | 19.4% | 15.3% | 11.7% | 15.5% | 11.6% |
| Admission to neonatal intensive care unit | 6.0% | 3.7% | 2.5% | 2.6% | 3.0% |
| Primary caesarean section | 13.3% | 8.2% | 10.1% | 7.5% | 6.4% |
| Secondary caesarean section | 9.2% | 8.0% | 3.5% | 8.4% | 7.0% |
| Pre-eclampsia/hypertension | 4.8% | 5.1% | 4.8% | 7.4% | 5.9% |
| Postpartum haemorrhage | 6.8% | 7.8% | 6.8% | 6.0% | 6.0% |
| No postpartum care (at home) | 11.2% | 9.1% | 7.8% | 4.7% | 5.3% |
| No antenatal care before wk 10 | 25.3% | 20.9% | 22.3% | 14.7% | 10.8% |
