# Supplementary material for: Defining vulnerability subgroups among pregnant women using pre-pregnancy information: a latent class analysis
Source: Eur J Public Health. 2022 Dec 14;33(1):25–34. doi: 10.1093/eurpub/ckac170 (PMC10263266; doi:10.1093/eurpub/ckac170)
Supplement: ckac170_Supplementary_Data [file ckac170_supplementary_data.zip › ckac170_Supplementary_Data/ejph-2022-06-om-0336-File010.docx]

Appendix 5. References (continued)

41. Wilson JMG, Jungner G, Organization WH. Principles and practice of screening for disease. 1968.

42. van Blarikom E, de Kok B, Bijma HH. “Who am I to say?” Dutch care providers' evaluation of psychosocial vulnerability in pregnant women. Soc Sci Med. 2022;307:115181.

43. Pearce A, Dundas R, Whitehead M, Taylor-Robinson D. Pathways to inequalities in child health. Archives of disease in childhood.Arch Dis Child. 2019;104(10):998-1003.
